# Sex differences in electrocardiographic patterns among patients with acute coronary syndromes

**DOI:** 10.3389/fcvm.2026.1758485

**Published:** 2026-10-12

**Authors:** Francesco Vitali, Giorgia Azzolini, Marco Zuin, Nicola Bianchi, Michele Malagù, Martina De Raffele, Anna Cantone, Federico Sanguettoli, Federico Gibiino, Cristina Balla, Elisabetta Tonet, Rita Pavasini, Gianluca Campo, Matteo Bertini

**Affiliations:** Cardiology Unit, Sant’Anna University Hospital, Department of Translational Medicine, University of Ferrara, Ferrara, Italy

**Keywords:** acute coronary syndrome (ACS), electrocardiagram (ECG), NSTEMI = non ST elevation myocardial infarction, sex differeces, STEMI (myocardial infarction)

## Abstract

Sex-based differences in ACS presentation and outcomes have been observed, but limited information is available on sex-specific electrocardiogram (ECG) features and their prevalence in this context. The aim of this study was to compare the prevalence of different ECG patterns between sexes and their prognostic roles for in-hospital mortality in ACS patients. This prospective single-center study (NCT02438085)° was conducted from June 2018 to December 2019, including 841 consecutive patients. Data on baseline characteristics, ECG findings, treatments, and outcomes were collected. Statistical analyses were performed to determine differences in the prevalence of various ECG patterns and identify independent predictors of in-hospital mortality. Males more frequently presented with ST-segment elevation (51% vs. 43%, *p* = 0.031) and hyperacute T waves (13% vs. 7%, *p* = 0.006) than females. Moreover, females more often had a normal ECG at admission (15% vs. 9%, *p* = 0.01). In female STEMI negative T waves (50% vs. 38%, *p* = 0.025) were more common than in males. Hyperacute T waves were linked to a higher risk of in-hospital death in female STEMI cases (OR=6.25, 95% CI: 1.0 −39.7, *p* = 0.048). Additionally the development of left bundle branch block during hospitalization (OR=14.4, 95% CI: 1.48–14 *p* = 0.02) was a significant predictor of in-hospital mortality in the female NSTEMI subgroup. No ECG features were found to predict in-hospital mortality in male patients. Compared to males, females with ACS often showed less frequent and less extensive ST-segment abnormalities and were more likely to have a normal initial ECG at admission. These differences could impact clinical evaluation and prognosis. Recognizing these sex-related ECG differences may help optimize the management of ACS patients.

**Clinical Trial Registration:** https://clinicaltrials.gov/ct2/show/NCT02438085

## Graphical Abstract

**Figure:**
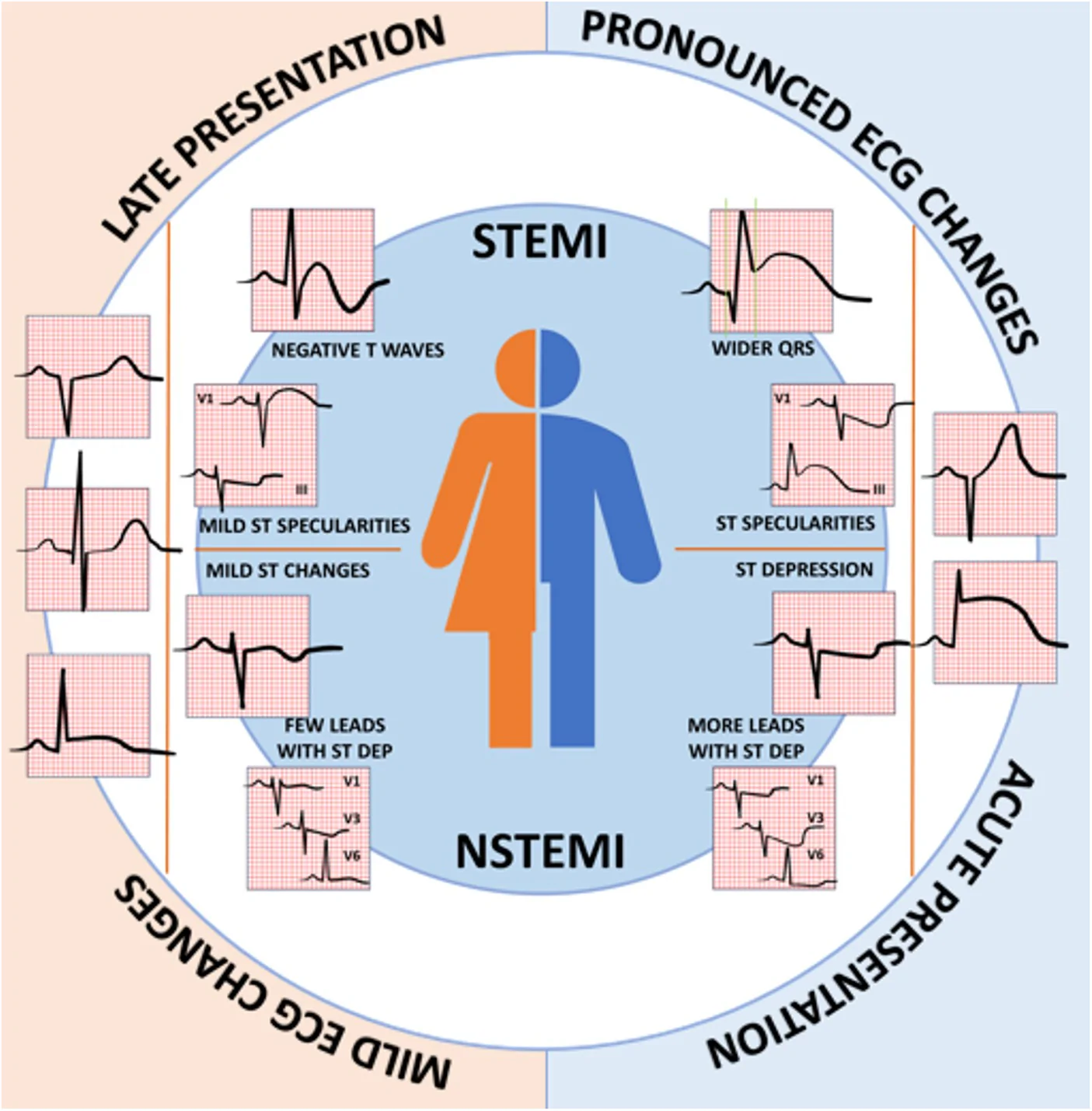
Inner circle – left part: ECG features specific to females in the STEMI subgroup include negative T wavespotentially linked to delayed presentation. In the NSTEMI subgroup, female patients exhibit mild ECG changes such as subtle ST segment alterations and isolated leads with ST segment depression. Outer circle – left part: female patients often present with initial ECGs showing mild changes or absence of repolarization abnormalities. Inner circle – right part: ECG features observed in male patients within the STEMI subgroup encompass broader QRS complexes and evident ST segment specularities. In the NSTEMI subgroup for males, there is a higher prevalence of ST segment depression, affecting multiple leads. Outer circle – right part: patients commonly present with hyperacute T waves and noticeable ST segment abnormalities in their initial ECGs.

## Introduction

Acute coronary syndrome (ACS) encompasses a range of conditions leading to myocardial ischemia and injury, including ST-segment elevation myocardial infarction (STEMI) and non-ST-segment elevation myocardial infarction (NSTEMI) (1, 2).

Females, especially at a younger age, have higher rates of NSTEMI and unstable angina (UA), whereas males more frequently present with STEMI (3). Regarding clinical presentation, females often exhibit atypical symptoms, which can lead to misdiagnosis (4). Females also commonly present later during myocardial infarction, resulting in delayed revascularization (5). Although it is well established that females derive the same benefits from percutaneous coronary intervention (PCI) as males, they receive fewer invasive investigations and reperfusion procedures, and are more likely to be treated conservatively (6, 7). While the electrocardiogram continues to play a central role in the initial evaluation and diagnosis of patients presenting with chest pain, scant information exists on sex-specific variations in ECG features among individuals experiencing ACS. The latest ESC guidelines on ACS (2) specify sex-specific STEMI criteria: for males, it is required to have a new ST elevation at J-points ≥ 2.5 mm if under 40 years old or ≥ 2 mm if 40 years old or older in leads V2-V3. For females, regardless of age, a new ST elevation ≥ 1 mm is required in leads V2-3; in all other leads, a deviation of ≥ 1 mm is required for both males and females. Sex-specific cut-offs in V2–V3 are deemed necessary due to generally lower J-point elevations in healthy women under resting conditions (8, 9). Moreover, international guidelines do not offer specific ECG sex criteria for NSTEMI (2). It is well known that many sex differences in cardiac electrophysiology exist, but studies on the relationship between electrocardiographic features and sex in the context of acute coronary syndromes are almost completely lacking. Furthermore, literature lacks data regarding the potential difference in the relationship between ECG features and mortality between males and females. The aim of this study was to assess sex differences in ECG features of patients admitted for ACS and to evaluate their correlations with the short-term prognosis.

## Methods

This analysis was conducted using a subset of the ARYOSTO study cohort**.** The Acute coRonary sYndrOmes proSpective regisTry Of Ferrara (ARYOSTO) (NCT n° NCT02438085) is a prospective, single-center study collecting data about baseline characteristics, treatment, and outcomes of all patients admitted to the University Hospital of Ferrara with a diagnosis of ACS (10, 11). The study is registered on clinicaltrials.gov with the identifier NCT02438085. The ARYOSTO study began in May 2015 and is currently ongoing. For the present analysis, we considered consecutive patients admitted to our center from June 2018 to December 2019. The study was conducted in accordance with the ethical principles of the Declaration of Helsinki and approved from our local ethic committee. Patients were informed that their participation was voluntary, and all gave written consent.

## Study population

Inclusion criteria were diagnosis of ACS (STEMI and NSTEMI) according to current European guidelines on the management and treatment of ACS (2). All ECGs were recorded and electronically stored. Patients were excluded from the analysis if they were diagnosed with other conditions mimicking an ACS, particularly those more prevalent among women, such as Takotsubo Syndrome, during their hospitalization. No other exclusion criteria were added. Patients unable to sign the Informed Consent were excluded. The management of participants was at the discretion of attending physicians and followed institutional protocols and international guidelines.

## Data collection

All clinical, ECG, treatment and outcome data were prospectively collected using a dedicated electronic case report form (eCRF) using the RedCap (Research Electronic Data Capture Consortium) platform. The eCRFs were periodically monitored and verified with source data. Specifically, the following data were prospectively collected: anthropometric data, cardiovascular (CV) risk factors, CV history and comorbidities, laboratory data, CV drugs administered during hospitalization, first ECG characteristics, procedural details including descriptions of the extension of coronary artery disease, interventional strategies, stent placements, procedural and management complications, and access site as well as in-hospital adverse events.

## Electrocardiography analysis

All ECGs recorded digitally throughout the patients’ hospitalization underwent analysis. ECGs were recorded at a standard speed of 25 mm/s and a voltage of 10 mm/mV. The initial presentation ECG at admission in the Emergency Department underwent analysis, and in cases where it showed no signs of ACS, we proceeded to examine the first positive ECG during hospitalization indicative of ACS recorded for each patient. Patient with new onset left bundle branch block (LBBB) or paced rhythm/known LBBB but with Sgarbossa criteria were included in the STEMI group (12). Two expert cardiologists (M. D. R. and R. P.), independently and blindly to sex and outcomes, conducted the ECG analysis, adhering to standard definitions and diagnostic criteria (13). ECG quantitative analysis was performed electronically, while qualitative analysis (such as the presence of hyperacute T wave) was conducted visually (M. D. R. and R. P.), and the presence of the ECG sign was accepted only if an agreement was reached. Each ECG analyzed was thoroughly assessed for all parameters including: heart rate (bpm), PR interval duration (ms), PR abnormalities, QRS duration in lead DII (ms), QRS axis, left ventricle hypertrophy, right bundle branch block, left bundle branch block, left anterior fascicular block, presence and localization of Q waves, QTc (ms), ST segment elevation entity (mm) and localization, number of leads with ST segment elevation, ST deviation specularity, presence of hyperacute T waves and localization, presence of negative T waves and localization, ST segment depression entity (mm) and localization, number of leads with ST segment depression, low peripheral potential, R/S > 1 in lead V1. In instances of disagreement, resolution occurred through consultation with a third senior cardiologist (M. M).

## Coronary angiography

The culprit lesion (infarct-related artery) was identified during coronary angiography based on a combination of angiographic findings (such as acute thrombus visualization, plaque rupture, filling defects, or abrupt vessel occlusion), electrocardiographic changes (ST-segment elevation/depression or T-wave inversions pointing to a specific vascular territory), and regional wall motion abnormalities on echocardiography. Significant obstructive CAD was defined as a lumen reduction of ≥70% in any of the three major epicardial coronary arteries (left anterior descending, left circumflex, or right coronary artery) or their main branches, and ≥50% for the left main coronary artery. Patients were classified as having single-vessel, two-vessel, or three-vessel disease based on the number of vessels involved.

## Study endpoints

The primary outcome was to estimate the prevalence and compare potential differences in the presentation ECG patterns between female and male patients with ACS. Additionally, as secondary outcome we aimed to explore their association with in-hospital mortality stratified according to the type of ACS.

## Statistical analysis

Continuous variables were expressed as mean ± standard deviation when normally distributed, as assessed using the Shapiro–Wilk test, or as median and interquartile range (IQR) for non-normally distributed variables. Categorical variables were summarized using frequency counts and percentages. Differences between male and female groups were assessed using unpaired t-tests for normally distributed continuous variables, Mann–Whitney U tests for non-normally distributed continuous variables, or Fisher's exact tests for categorical variables.

To analyze risk factors for in-hospital mortality, the population was stratified into four subgroups based on STEMI or NSTEMI presentation and sex. Univariate logistic regression was performed for each potential risk factor. A multivariate logistic regression model was then developed based on the significant factors identified in the univariate analysis. Statistical significance was defined as a *p*-value < 0.05.

All statistical analyses were performed using Stata/SE version 16 software (StataCorp, College Station, TX, USA).

## Results

A total of 841 patients were enrolled in this study, of which 550 were male (65%). The baseline clinical characteristics are summarized in Table 1. The median age was 70 years (IQR 60–80) and males were younger than females (68; IQR 58–77 vs. 76; IQR 66–83; *p* < 0.001). Regarding cardiovascular risk factors, males were more frequently smokers (61% vs. 28%, *p* < 0.001), had a higher BMI (27.0 vs. 25.9, *p* = 0.006) and a family history of CAD (15 vs. 8.3%, *p* = 0.005), while females more frequently had hypertension (78% vs. 67%, *p* < 0.001). Males had a higher frequency of a history of prior myocardial infarction (26% vs. 18%, *p* = 0.008), as well as prior reperfusion both through PCI (24% vs. 14%, *p* = 0.002) and CABG (7.3% vs. 3.8%, *p* = 0.049). In terms of presentation, there was no significant difference in the incidence of cardiac arrest (2.5% vs. 1.4%, *p* = 0.32) between the two groups. Almost all enrolled patients presented within 3 h from the onset of symptoms, with female patients tending to present later from symptom onset (178 min IQR 100–394 vs. 219 min IQR 110–445; *p* = 0.042). Coronary angiography was performed in 98% of cases, with no differences between males and females (99% vs. 97%, *p* = 0.17) while PCI was more frequently performed in males than in females (88% vs. 71%, *p* < 0.001). None of the study population underwent CABG. The main differences in coronary anatomy were as follows: in males, the most common location of the culprit lesion was in the left anterior descending artery (37% vs. 32%), while in females, multiple lesions without a clear culprit were more common (17% vs. 34%); males tended to have more frequent three-vessel disease with (19% vs. 11%) or without (10% vs. 6.9%) involvement of the left main coronary artery, whereas non-significant coronary artery disease was a more common finding in females (9.3% vs. 29%). In our cohort, we observed no significant sex-related difference in the choice of P2Y12 inhibitor among STEMI patients. Prasugrel was prescribed in 23/119 (19.3%) women and 63/268 (23.5%) men, whereas ticagrelor was prescribed in 96/119 (80.7%) women and 205/268 (76.5%) men (*p* = 0.435). In NSTEMI patients, a statistically significant difference in the distribution of P2Y12 inhibitor therapy was observed between sexes (*p* = 0.01), with women receiving clopidogrel more frequently and men receiving ticagrelor more frequently, while prasugrel use was low in both groups.

**Table 1:** Baseline characteristics of the study population.

	Male (550 patients)	Female (291 patients)	p
Age (years)	68 (58–77)	76 (66–83)	<0.001
BMI (kg/m^2^)	27 ± 4.7	25.9 ± 6.3	0.006
Hypertension, *n* (%)	369 (67%)	226 (78%)	<0.001
Dyslipidemia, *n* (%)	266 (48%)	133 (46%)	0.56
Smokers, *n* (%)	337 (61%)	81 (28%)	<0.001
Diabetes mellitus, *n* (%)	136 (25%)	70 (24%)	0.93
Family history of CAD, *n* (%)	83 (15%)	24 (8.3%)	0.005
Prior myocardial infarction, *n* (%)	145 (26%)	52 (18%)	0.008
Prior PCI, *n* (%)	131 (24%)	42 (15%)	0.002
Prior CABG, *n* (%)	40 (7.3%)	11 (3.8%)	0.049
Prior atrial fibrillation or flutter, *n* (%)	56 (10%)	30 (10%)	1.00
Stroke, *n* (%)	37 (6.7%)	11 (3.8%)	0.09
TIA, *n* (%)	12 (2.2%)	10 (3.5%)	0.27
Active cancer, *n* (%)	30 (5.5%)	17 (5.9%)	0.87
Anemia, *n* (%)	22 (4%)	17 (5.9%)	0.23
COPD, *n* (%)	29 (5.3%)	17 (5.9%)	0.75
Chronic kidney disease, *n* (%)	64 (12%)	29 (10%)	0.56
Presentation and procedural aspects			
STEMI	268 (48,7%)	119 (40,9%)	0,03
NSTEMI	282 (51,3%)	172 (59,1%)	0,03
Cardiac arrest, *n* (%)	14 (2.5%)	4 (1.4%)	0.32
Troponin-I high sensitivity peak value	3,188 (326–16,354)	2,342 (294–14,590)	0.33
Time from the symptom onset (min) • All • STEMI • NSTEMI	178 (100–394) 175 (112–394) 180 (94–372)	219 (110–445) 204 (114–439) 238 (89–445)	0.042 0.24 0.13
Coronary angiography performed, *n* (%)	266 (99%)	115 (97%)	0.17
PCI performed, *n* (%)	237 (88%)	84 (71%)	<0.001
Culprit lesion, *n* (%) • LAD • LM • Cx • RCA • Bypass • Multiple/unkown	206 (37%) 23 (4.2%) 86 (16%) 134 (24%) 8 (1.4%) 93 (17%)	93 (32%) 9 (3.1%) 35 (12%) 51 (18%) 2 (0.7%) 100 (34%)	<0.001
N. of diseased vessels, *n* (%) • One • Two • Three without LM • Three with LM • Non significat CAD/normal CA	179 (32%) 156 (28%) 107 (19%) 57 (10%) 51 (9.3%)	95 (33%) 60 (21%) 32 (11%) 20 (6.9%) 84 (29%)	<0.001
ECG characteristics			
Heart rate (bpm)	78 (64–93)	79 (67–97)	0.16
PR interval (ms)	160 (1,44,182)	160 (142–180)	0.36
Advanced AVB, *n* (%)	30 (5%)	7 (2%)	0.042
QRS duration (ms)	98 (90–110)	90 (82–102)	<0.001
ST segment elevation	268 (51%)	118 (43%)	0.03
QTc (ms)	433 (410–461)	444 (427–469)	<0.001
Hyperacute T waves	70 (13%)	19 (7%)	0.006
First medical contact normal ECG	48 (9%)	43 (15%)	0.01

AVB, atrio-ventricular block; BMI, body mass index; CAD, coronary artery disease; PCI, percutaneous coronary intervention; CABG, coronary artery bypass graft; TIA, transient ischemic attack; COPD, chronic obstructive pulmonary disease; LM, left main; LAD, left anterior descending artery; Cx, circumflex artery; RCA, right coronary artery; CA, coronary arteries; bpm, beats per minute; ms, milliseconds.

## Electrocardiographic analysis

Regarding ECG patterns, no differences were observed in terms of heart rate, PR interval and its anomalies. The median QRS duration was 94 ms (IQR 86–108 ms), which was higher in males than in females (98 ms; IQR 90–110 ms vs. 90 ms; IQR 82–102 ms) and the QTc interval was shorter in males than females (433 ms; IQR 410–461 ms vs. 444; IQR 427–469 ms) as expected. As for cardiac rhythms, no difference was observed in the incidence of atrial fibrillation or flutter and 80% of the entire population was in sinus rhythm.

## Primary endpoint

In our study population, males presented with ST segment elevation more frequently than females (51% vs. 43%, *p* = 0.031), as well as hyperacute T waves (13% vs. 7.0%, *p* = 0.006). On the other hand, females more frequently had a first medical contact normal ECG (15% vs. 9%, *p* = 0.01), except for QTc duration, which was longer than in males (444 ms vs. 433 ms, *p* < 0.001), in both the STEMI and NSTEMI subgroups. (Table 2).

**Table 2:** ECG features in ST-segment elevation myocardial infarction (STEMI) and in non ST-segment elevation myocardial infarction (NSTEMI) subgroups.

STEMI					
	Male (268 patients)			Female (119 patients)	p
Heart rate (bpm)	79 (64–95)			82 (69–100)	0.14
PR interval (ms)	160 (144–186)			156 (140–176)	0.12
QRS duration (ms)	98 (90–106)			90 (82–100)	<0.001
Right bundle branch block	17 (6.4%)			11 (9.3%)	0.30
Left bundle branch block	5 (1.9%)			1 (0.8%)	0.67
QTc (ms)	428 (406–454)			435 (422–461)	0.022
ST segment elevation entity (mm)	2 (2–3)			2 (2–3)	0.053
Number of leads with ST segment elevation	3 (2–4)			3 (2–4)	0.62
ST deviation specularity	140 (54%)			49 (43%)	0.044
Hyperacute T waves	56 (21%)			15 (13%)	0.063
Negative T waves	100 (38%)			59 (50%)	0.025
NSTEMI					
		Male (282 patients)	Female (172 patients)		p
Heart rate (bpm)		78 (64–92)	78 (67–93)		0.47
PR interval (ms)		160 (144–180)	162 (144–184)		0.96
QRS duration (ms)		98 (88–118)	90 (80–102)		<0.001
Right bundle branch block		10 (3.7%)	4 (2.4%)		0.58
Left bundle branch block		27 (10%)	20 (12%)		0.53
QTc (ms)		436 (416–465)	450 (432–473)		<0.001
ST segment depression		85 (32%)	30 (19%)		0.003
Number of leads with ST segment depression		1 (0–3)	0 (0–1)		0.01
Hyperacute T waves		14 (5.4%)	4 (2.5%)		0.22
Negative T waves		77 (30%)	38 (24%)		0.28
First medical contact normal ECG		48 (18%)	43 (26%)		0.052

## Electrocardiographic sex-differences STEMI subgroup

In the STEMI subgroup, no differences were observed between males and females in terms of incidence and entity of ST deviation, ST elevation localization, the number of involved leads, the frequency of hyperacute T waves, low QRS voltage, new onset bundle branch blocks (both right and left) or hemiblocks. In women negative T waves (50% vs. 38%, *p* = 0.025) were observed more frequently. Additionally, males had longer QRS duration (98 ms; IQR 90–106 vs. 90 ms; IQR 82–100, *p* < 0.001) and more frequently ST deviation specularities (54% vs. 43%, *p* = 0.044) than females. There were no differences observed in the localization of myocardial infarction between males and females (Table 2).

## Electrocardiographic sex-differences for NSTEMI subgroup

In the NSTEMI subgroup the presence of ST depression was statistically more frequent in the male population than in the female population (32% vs. 19%, *p* = 0.003). The number of leads involved by ST segment deviation was statistically different between males and females with males presenting with ST depression in more leads (1; IQR 0–3 vs. 0; IQR 0–1, *p* = 0.011) (Table 2).

## In hospital mortality and associated ECG features

Overall, 5.5% (*n* = 46) of the population died in-hospital. No significant differences in terms of mortality were found between males and females (*p* = 0.75), neither in the STEMI (3.7% vs. 7.6%, *p* = 0.13) nor in the NSTEMI subgroups (6.7% vs. 4.7%, *p* = 0.42).

In the STEMI subgroup only age among clinical parameters was associated with an increased risk of in-hospital death in both males (OR 1.1; CI 1.03–1.19) and females (OR 1.15; CI 1.03–1.27). Moreover, in the female STEMI subgroup, hyperacute T waves were also found to be associated with increased risk of in-hospital death with OR of 6.25 (1.0–39.7 *p* = 0.048).

From the logistic regression analysis of in-hospital mortality, in the female NSTEMI subgroup development of left bundle branch block during hospitalization was associated with an increased risk of in-hospital death, with odd ratio of 14.4 (1.48–14.0, *p* = 0.02) respectively. On the other hand, this correlation was not observed in the male NSTEMI subgroup (Table 3).

**Table 3:** Multivariable regression analysis of clinical and ECG features associated with in-hospital mortality in different study subgroups.

	OR	CI95%	p
NSTEMI Male			
Heart rate	1.02	1.00–1.04	0.055
Atrial fibrillation	2.72	0.65–11.4	0.173
Ventricular arrhythmias	13.1	0.75–2.27	0.078
NSTEMI Female			
Heart rate	1.04	0.99–1.07	0.06
Development LBBB during hospitalization	14.4	1.48–140	0.02
STEMI Male			
Age	1.10	1.03–1.19	0.007
QRS duration	1.03	1–1.05	0.069
QTc interval duration	1	0.99–1.01	0.5
STEMI Female			
Age	1.15	1.03–1.27	0.012
Low QRS potentials	13.8	1.53–129	1.5
Hyperacute T waves	6.25	1.0–39.7	0.048

## Discussion

Despite its central role in the diagnostic process of ACS, the literature lacks data on the relationship between ECG features and sex. ECG is inexpensive and widely performed; understanding the prognostic role of certain ECG signs in both sexes can help identify which patients require closer monitoring after the revascularization procedure and determine the appropriate hospital setting for their admission. Our major findings are as follows: (1) in the context of ACS, females tend to present with only mild electrocardiographic changes; (2) females more frequently exhibited a first normal ECG upon admission; (3) in the STEMI subgroup, female more frequently exhibit negative T waves, which are likely related to their delayed presentation; (4) there was no significant difference in in-hospital mortality between males and females (Graphical Abstract). However, among female NSTEMI patients, the presence of new onset left bundle branch block during the hospital stay (OR 14.4; CI 95% 1.48–14) was associated with an increased risk of in-hospital mortality. Similarly, hyperacute T waves (OR 6.25; CI 95% 1.0–39.7) were associated with increased risk of in-hospital mortality among female STEMI subgroup.

It is already well known that some dissimilarities in electrocardiographic features exist between males and females, which could potentially play a role in the management of clinical conditions. Understanding these differences could lead to optimized patient management. At baseline, differences can be found in mean sinus rate, QRS duration and amplitude, QT interval, J point and ST segment and T wave (14).

When considering QRS complexes, our results reveal that they were consistently longer in men than in women in both the STEMI and NSTEMI subgroups, which aligns with data from the general population (15).

Both J point and ST segment amplitude are typically higher in healthy adult males compared to age-matched females (16) (Figures 1, 2). The last ESC guidelines for the management of acute myocardial infarction in patients presenting with ST-segment elevation (2) consider these aspects, providing specific age- and sex-related cut-off criteria. In the subgroup of the study population which presented with ST segment elevation (STEMI subgroup), we observed no significant differences in terms of the extent and number of leads with ST deviation. However, specularity alterations were observed more often in males than in females. In NSTEMI subgroup males tend to present more frequently with ST segment depression than females. These findings again support the supposed generally less frequent or less extensive ST-segment abnormalities in female's electrocardiograms, potentially making their initial clinical evaluation more challenging.

**Figure 1:**
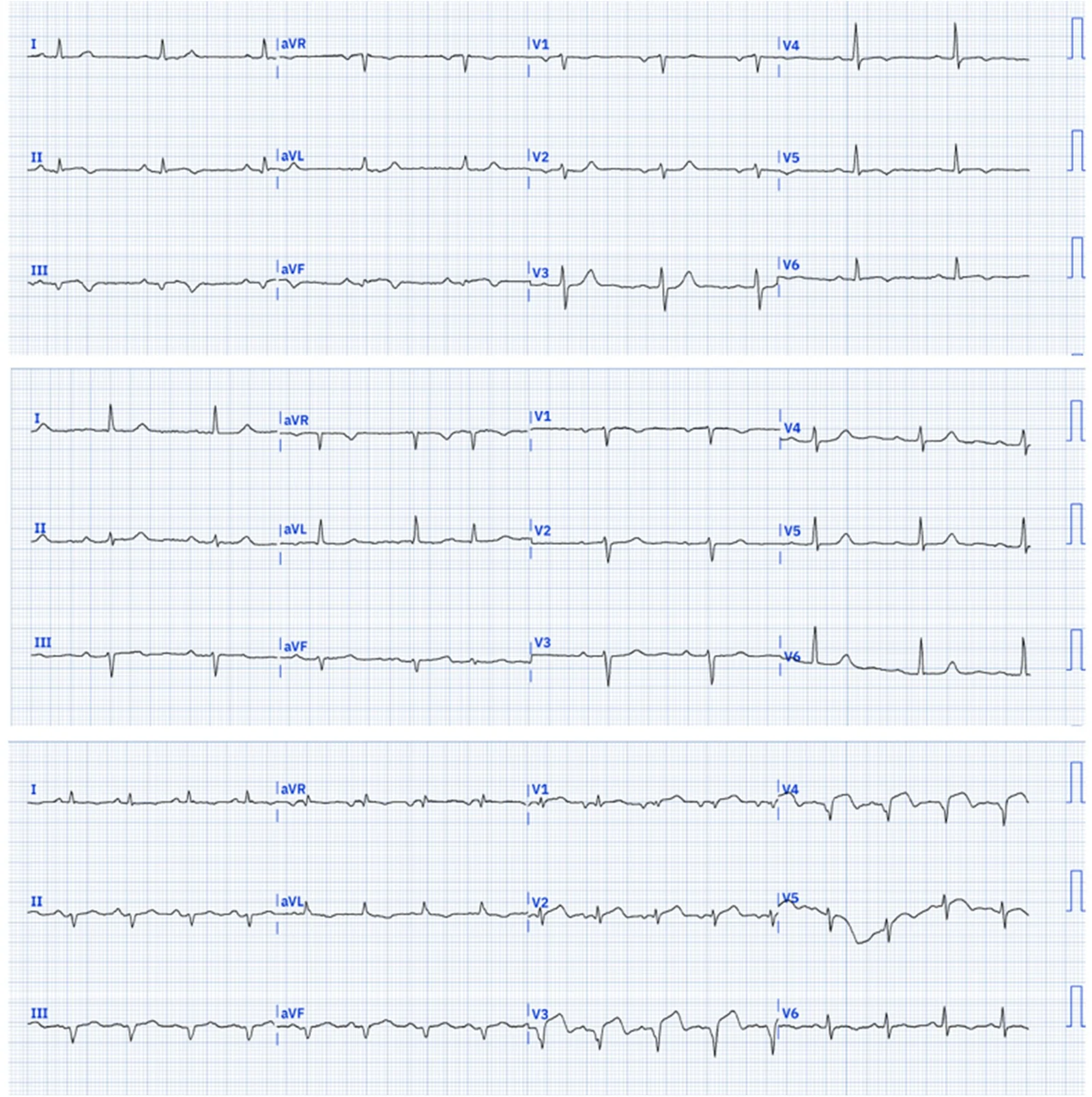
Females ECG examples, from top to bottom: first ECG: Sinus rhythm with mild negative T waves and slight ST segment elevation in inferior and lateral leads. Second ECG: Sinus rhythm and mild ST segment depression in lateral leads. Third ECG: Sinus tachycardia with ST segment elevation and Q waves in the anteroseptal leads.

**Figure 2:**
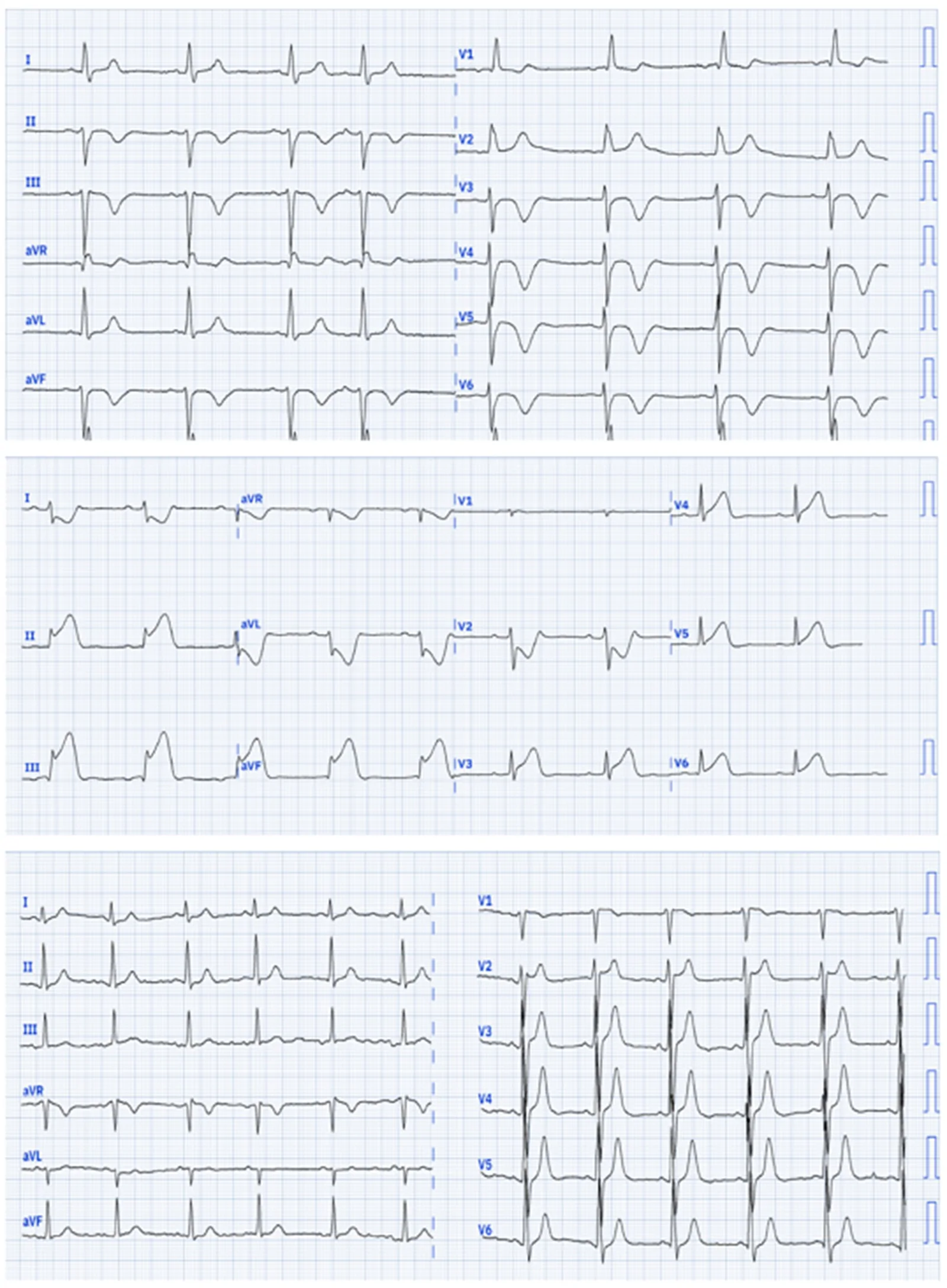
Males ECG examples, from top to bottom: first ECG: sinus bradycardia with right bundle branch block and QRS left axis deviation with deep negative T waves in the precordial leads. Second ECG: sinus bradycardia with ST segment elevation in inferior and lateral leads with evident ST segment specularities in anterolateral leads. Third ECG: sinus rhythm with hyperacute T wave in the precordial leads.

Baseline T waves have lower amplitude and longer duration in females compared to males, as evidence of a slower repolarization mechanism (8, 17). We observed negative T waves more frequently in females than in males in the STEMI subgroup, but not in the NSTEMI one. Negative T waves could serve as another marker of delayed presentation of females as mentioned above. In contrast, hyperacute T waves were observed more frequently in males than in females, further highlighting the sex-related differences in presentation time.

The QT interval is longer in females than in males, as observed and confirmed by further studies (8). We observed longer QTc intervals in females in both the STEMI and NSTEMI subgroups. This difference could be relevant in relation to the greater susceptibility to life-threatening arrhythmias linked to a longer QTc interval.

From the logistic regression analysis of in-hospital mortality, we observed an increased risk of in-hospital death in the context of NSTEMI in the female subgroup in association with development of left bundle branch block during the hospitalization. Additionally, in the context of STEMI, females tend to have poorer prognosis, namely increased in-hospital death, if they presented themselves with hyperacute T waves. In contrast, no mortality predictors related to ECG features were found in male patients, neither in the STEMI nor in the NSTEMI subgroups, with only age being correlated with increased risk of in-hospital mortality.

The present findings warrant validation in independent multicenter cohorts to confirm the reproducibility and generalizability of the observed sex-specific ECG patterns. In particular, larger studies with standardized ECG acquisition protocols could help clarify whether the prognostic value of specific ECG features differs between men and women. Furthermore, future research should explore the role of subtle and dynamic ECG changes during the early phase of ACS presentation, which may be particularly relevant in female patients who more frequently present with non-diagnostic initial ECGs.

## Limitations

This study has some limitations, first and foremost it is a single-center study, which may limit the generalizability of the findings to a broader population, potentially not fully representing the diversity of patient demographics, healthcare practices and regional variations. The enrollment of patients was limited as a result of the need to meticulously analyze the ECGs conducted for each individual. Consecutive enrollment of ACS patients presents significant logistical challenges, particularly with regard to patients not admitted to a cardiology ward, often comprising the most frail and elderly patients, or those who succumb before admission/consent process, representing the highest-risk group. ACS is an extremely dynamic condition, so certain ECG signs may have appeared later during hospitalization and for this reason some patients were non included in the analysis. When the initial ECG was normal, the first ECG showing diagnostic abnormalities during hospitalization was used for analysis. Since women more frequently presented with a normal initial ECG, the timing of ECG assessment may have differed between sexes, potentially reflecting different stages of disease progression. A detailed mechanistic classification of ACS phenotypes (e.g., plaque rupture, plaque erosion, vasospasm) was not systematically available, as intracoronary imaging with Optical Coherence Tomography (OCT) or Intravascular Ultrasound (IVUS) was not routinely performed. Another limitation is the relatively small number of in-hospital deaths observed in the study. Consequently, the multivariable analyses may have been underpowered to fully adjust for all potential confounders, and the resulting effect estimates should be interpreted with caution. Therefore, these findings should be considered exploratory and hypothesis-generating and warrant confirmation in larger cohorts with a greater number of outcome events. Finally, sex-related differences in antiplatelet therapy prescription, particularly in NSTEMI patients, could have acted as residual confounders. Because the study was not designed or powered to evaluate the impact of specific antiplatelet regimens on outcomes, this potential influence cannot be completely excluded.

## Conclusion

In the context of acute coronary syndrome, females tend to have less frequent or less extensive ST-segment abnormalities in both STEMI and NSTEMI in comparison to males. Consequently, this can make the initial evaluation of female patients presenting with symptoms of SCA more challenging. Furthermore, certain ECG features were found to be associated with poorer prognosis in female patients, underscoring the importance of giving proper consideration of even less frequent and less extensive ST-segment abnormalities in this subgroup of population.

## Data Availability

The raw data supporting the conclusions of this article will be made available by the authors, without undue reservation.

## References

[1] Roth GA Johnson C Abajobir A Abd-Allah F Abera SF Abyu G. Global, regional, and national burden of cardiovascular diseases for 10 causes, 1990 to 2015. J Am Coll Cardiol. (2017) 70(1):1–25. 10.1016/j.jacc.2017.04.052 28527533 PMC5491406

[2] Byrne RA Rossello X Coughlan JJ Barbato E Berry C Chieffo A. 2023 ESC guidelines for the management of acute coronary syndromes. Eur Heart J. (2023) 44(38):3720–826. 10.1093/eurheartj/ehad191 37622654

[3] Akhter N Milford-Beland S Roe MT Piana RN Kao J Shroff A. Gender differences among patients with acute coronary syndromes undergoing percutaneous coronary intervention in the American College of Cardiology-National Cardiovascular Data Registry (ACC-NCDR). Am Heart J. (2009) 157(1):141–8. 10.1016/j.ahj.2008.08.012 19081410

[4] Hochman JS Tamis JE Thompson TD Weaver WD White HD Van de Werf F. Sex, clinical presentation, and outcome in patients with acute coronary syndromes. Global use of strategies to open occluded coronary arteries in acute coronary syndromes IIb investigators. N Engl J Med. (1999) 341(4):226–32. 10.1056/NEJM199907223410402 10413734

[5] Stehli J Martin C Brennan A Dinh DT Lefkovits J Zaman S. Sex differences persist in time to presentation, revascularization, and mortality in myocardial infarction treated with percutaneous coronary intervention. J Am Heart Assoc. (2019) 8(10):e012161. 10.1161/JAHA.119.012161 31092091 PMC6585344

[6] Radovanovic D Erne P Urban P Bertel O Rickli H Gaspoz JM. Gender differences in management and outcomes in patients with acute coronary syndromes: results on 20,290 patients from the AMIS plus registry. Heart. (2007) 93(11):1369–75. 10.1136/hrt.2006.106781 17933995 PMC2016893

[7] Nguyen HL Goldberg RJ Gore JM Fox KA Eagle KA Gurfinkel EP. Age and sex differences, and changing trends, in the use of evidence-based therapies in acute coronary syndromes: perspectives from a multinational registry. Coron Artery Dis. (2010) 21(6):336–44. 10.1097/MCA.0b013e32833ce07c 20661139

[8] Surawicz B Parikh SR. Differences between ventricular repolarization in men and women: description, mechanism and implications. Ann Noninvasive Electrocardiol. (2003) 8(4):333–40. 10.1046/j.1542-474X.2003.08411.x 14516290 PMC7313228

[9] Van Noord C Dörr M Sturkenboom MC Straus SM Reffelmann T Felix SB. The association of serum testosterone levels and ventricular repolarization. Eur J Epidemiol. (2010) 25(1):21–8. 10.1007/s10654-009-9406-z 19957021 PMC2807939

[10] Cimaglia P Fortini F Vieceli Dalla Sega F Cardelli LS Massafra RF Morelli C. Relationship between PCSK9 and endothelial function in patients with acute myocardial infarction. Nutrition, Metabolism, and Cardiovascular Diseases: NMCD. (2022) 32(9):2105–11. 10.1016/j.numecd.2022.06.020 35915019

[11] Pavasini R Fabbri G Marchini F Bianchi N Deserio MA Sanguettoli F. Procalcitonin predicts bacterial infection, but not long-term occurrence of adverse events in patients with acute coronary syndrome. J Clin Med. (2022) 11(3):554. 10.3390/jcm11030554 35160005 PMC8836946

[12] Sgarbossa EB Pinski SL Barbagelata A Underwood DA Gates KB Topol EJ. Electrocardiographic diagnosis of evolving acute myocardial infarction in the presence of left bundle-branch block. GUSTO-1 (global utilization of streptokinase and tissue plasminogen activator for occluded coronary arteries) investigators. N Engl J Med. (1996) 334(8):481–7. 10.1056/NEJM199602223340801 8559200

[13] Surawicz B Knilans T. Chou’s Electrocardiography in Clinical Practice. 6th edn. Amsterdam: Saunders (2008).

[14] Kittnar O. Sex related differences in electrocardiography. Physiol Res. (2023) 72(Suppl 2):S127–35. 10.33549/physiolres.934952 37565417 PMC10660582

[15] Hnatkova K Smetana P Toman O Schmidt G Malik M. Sex and race differences in QRS duration. Europace. (2016) 18:1842–49. 10.1093/europace/euw065 27142220

[16] Attia ZI Friedman PA Noseworthy PA Lopez-Jimenez F Ladewig DJ Satam G. Age and sex estimation using artificial intelligence from standard 12-lead ECGs. Circ Arrhythm Electrophysiol. (2019) 12(9):e007284. 10.1161/CIRCEP.119.007284 31450977 PMC7661045

[17] Gambill CL Wilkins ML Haisty WK Anderson ST Maynard C Wagner NB. T wave amplitudes in normal populations. Variation with ECG lead, sex and age. J Electrocardiol. (1995) 28:191–97. 10.1016/S0022-0736(05)80257-2 7595121

